# Moderate altitude exposure impacts extensive host-microbiota multi-kingdom connectivity with serum metabolome and fasting blood glucose

**DOI:** 10.1080/21505594.2025.2530660

**Published:** 2025-07-09

**Authors:** Xiaoran Huang, Xiaoyan Gao, Yanqun Fan, Dingchen Wang, Xuanfu Chen, Xin Qi, Zhibo Yang, Yu-E Wang, Jinxiu Meng, Guoxiang Zou, Zhipeng Liu, Xin Li

**Affiliations:** a Department of Emergency Medicine, Guangdong Provincial People’s Hospital (Guangdong Academy of Medical Sciences), Southern Medical University, Guangzhou, China; b Department of General Practice, Nyingchi People’s Hospital, Tibet, China; c Department of Trans-omics Technology Research and Development, Biotree Metabolomics Technology Research Center, Shanghai, China; d School of Medicine, South China University of Technology, Guangzhou, China; e Guangdong Cardiovascular Institute, Guangdong Provincial People’s Hospital (Guangdong Academy of Medical Sciences), Guangzhou, China

**Keywords:** Moderate altitude, multi-kingdom microorganisms, serum metabolome, fasting blood glucose

## Abstract

The contributions and interactions of multi-kingdom microbiota (i.e. bacteriome, mycobiome, archaeome, and phageome) with serum metabolome and host phenome in healthy individuals under moderate altitude exposure remain unclear. We applied shotgun metagenomic sequencing in feces and targeted metabolomics technology in serum to explore how human gut multi-kingdom microorganisms influence the serum metabolome and phenome in healthy Chinese individuals following moderate altitude exposure. The results indicated that individuals with moderate altitude exposure exhibited more substantial alterations in gut bacteriome and phageome compared to those in mycobiome and archaeome. Both intra-kingdom and inter-kingdom correlations at baseline were denser than those following moderate altitude exposure. Bacteriophages-host interaction analysis revealed symbiosis between bacteriophages and *Bacteroidetes*, *Proteobacteria*, and short-chain fatty acids (SCFAs) producers. Furthermore, bacteriophage *Shirahamavirus PTm1* (odds ratio (OR) = 3.82; 95% confidence interval (CI): 1.20–12.16), archaeon *Crenarchaeota* (OR = 3.70; 95% CI: 1.35–10.14) and bacterium *Bacteroidetes* (OR = 3.69; 95% CI: 1.34–10.15) showed a positive association with lowered fasting blood glucose (FBG) benefits, while bacteriophage *Candidatus Nitrosopelagicus brevis* (OR = 0.30; 95% CI: 0.10–0.89) and butyric acid (OR = 0.07; 95% CI: 0.01–0.37) exhibited a negative association with lowered FBG benefits. These findings suggest that targeting gut multi-kingdom microorganisms could serve as an alternative therapeutic approach to mitigate dysglycemia and its associated metabolic disorders.

## Introduction

Moderate altitude is defined as 2000–3000 m, while high-altitude is classified as 3000–5500 m according to the previous consensus statement [1]. The natural moderate altitude or high-altitude environment is characterized by decreased barometric pressure, hypoxia, low temperature and increased ultraviolet radiation [2]. Increased heart ratio, hyperventilation and augmented red cell blood mass are well-known physiological responses in the humans acclimatizing to these challenging conditions [3]. Altitude has some beneficial effects on human physiology, such as an inverse relationship with adiposity and diabetes [4–7]. According to previous studies, individuals who underwent intermittent hypoxia exposure or made trips to high altitude mountains for short-term residence (3−21 days) experienced some improvements in FBG levels, weight loss or cardiopulmonary function [8–12]. Therefore, it is crucial to understand the precise role and underlying mechanisms of effects of high altitude on human health.

In recent decades, the importance of the microbiome to human health has gained increasing recognition and has become the primary focus of contemporary research. Human-associated microbiota, which comprise communities of bacteria, fungi, archaea, and viruses that inhabit the human body, plays a crucial role in shaping the human health. Dysbiosis of gut bacteria has been implicated in initiating or promoting the pathogenesis of various diseases, including cardiovascular disease, adiposity, type 2 diabetes (T2DM), neurodegenerative diseases and cancers [13–17]. Although previous studies on human microbiota primarily focused on the bacteria, the important roles of commensal fungi, archaea and viruses in human health are becoming increasingly evident [18–23]. All these components interact with each other and with the host to influence the overall health of the host. For example, the enriched *Saccharomycetales spp* may increase the abundance of SCFAs-producing bacteria, leading to enhanced production of SCFAs in the human gut, potentially improving insulin sensitivity [24, 25]. The host-associated bacteriome interacts with the archaeome by providing substrates (e.g. trimethylamine and H_2_), thereby influencing the production of carcinogens H_2_S and trimethylamine N-oxide [26, 27]. Archaeal methanogens also synergistically interact with bacteria to enhance the production of SCFAs [28] to suppress cancer development or produce other archaeal metabolites (e.g. indoles and secondary bile acids) that directly or indirectly participate in the initiation or progression of carcinogenesis [29]. Dysbiosis of gut bacteriophages (bacterial viruses shaping microbial communities) was found to be associated with metabolic syndrome, insulin resistance, obesity, gastroenterological diseases, and chronic social stress. These conditions can be ameliorated by fecal virome transplantation (FVT) from healthy donors [30–35]. Similar to FVT, fecal microbiota transplantation (FMT) and lifestyle interventions can restore gut microbiota homeostasis and improve health outcomes in patients with cardiometabolic diseases [36–41].

Recent investigations on gut multi-kingdom microorganisms in humans have expanded our knowledge of the gut microbiota system. Previous research has examined the landscape of the gut bacteriome, mycobiome, archaeome, and phageome in relation to geography, urbanization, ethnicity, and diet in the Chinese population spanning six ethnicities: Han, Zang, Bai, Hani, Dai, and Miao (including both urban and rural residents for each ethnicity) [25, 42, 43]. These studies demonstrated that geography exerted the strongest influence on the composition of gut archaeome and phageome, while urbanization had the most significant impact on the composition of gut bacteriome and mycobiome. Furthermore, studies on gut multi-kingdom microorganisms have enhanced our understanding of the pathophysiological mechanisms and microbial interactions in colorectal cancer, decompensation in outpatients with cirrhosis, and COVID-19 outcomes [44–46]. Diagnostic tools based on the analysis of multi-kingdom microorganisms could refine predictions and provide therapeutic targets to prevent disease progression. High-altitude exposure ranging from 1 day to 12 months has been reported to modulate the gut bacteria in animals (e.g. mice and yak) and humans, thereby influencing the host phenotype [40, 47–52]. However, the functional roles of gut multi-kingdom microorganisms and their contributions to host health following moderate altitude exposure for a long term (~12 months) have not been characterized. Therefore, studies on multi-kingdom microorganisms are urgently required to explore the biologically plausible mechanisms of the effects of moderate altitude exposure on human gut microbiota.

To address the above-mentioned knowledge gaps, we recruited 47 healthy Han Chinese individuals from Guangzhou city (average altitude =  <50 m) who relocated to Nyingchi city (average altitude = 2,900 m) for 12 months of residence, aiming to evaluate the impacts of moderate altitude exposure on the host multi-kingdom microorganisms, serum metabolome and phenome. Additionally, we enrolled 36 healthy Han Chinese and 11 healthy Tibetans residing in Nyingchi for over 5 years as a pilot study to examine the effects of ethnicity and prolonged moderate altitude exposure time on the host. We conducted shotgun metagenomics on fecal samples to depict the landscape of multi-kingdom microorganisms, bacteriophage-bacterial host interactions, and correlations between intra-kingdom and inter-kingdom microorganisms in individuals exposed to moderate altitude. We also performed an association analysis to explore the correlations among gut microbiota composition, serum metabolites (i.e. amino acids (AAs), fatty acids (FAs, including SCFAs and medium-chain fatty acids (MCFAs)), and bile acids (BAs)), and phenome in individuals exposed to moderate altitude.

## Materials and methods

### Study cohort, samples collections and biochemical measurements

The study was conducted in Nyingchi city (29.5° N, 94.3° E), known for its pleasant environment (http://www.linzhi.gov.cn/linzhi/zmlz/qh.shtml) and tourism-driven economy [53]. Detailed meteorological data of Nyingchi city are described in the supplementary methods. Eligible healthy volunteers were selected through disease inquiry and physical examination. None of the study subjects had taken hormones, anti-obesity agents, antibiotics, or probiotics at least 1 month before the initiation of the study. The study enrolled 94 healthy individuals assigned to two parts: (1) 47 healthy Han Chinese aid-Tibet volunteers (male/female = 35/12; age, 40.37 ± 6.06 years, mean ± s.d.) from Guangzhou city who entered Nyingchi city in July 2018 for 12-month stay. Anthropometric data (including body weight (BW), height, and body mass index (BMI), waist circumference (WC), systolic blood pressure (SBP), diastolic blood pressure (DBP), heart ratio (HR)) and blood samples were collected at baseline, months 6 and 12 post-moderate altitude exposure. Fecal samples were collected from individuals at baseline and months 12 after moderate altitude exposure. Some volunteers discontinued participation due to work commitments. (2) A long-term population (residing in Nyingchi for over 5 years) comprising 36 healthy Han Chinese population (group Local_H) (male/female = 10/26; age, 30.86 ± 8.30 years) and 11 healthy Tibetans population (group Local_Z) (male/female = 1/10; age, 35.73 ± 8.57 years). Blood samples, fecal samples, and anthropometric data were also collected from this group. To evaluate the dietary intake and physical activity of these volunteers before and after moderate altitude exposure, the self-reported food frequency questionnaires (FFQ) and physical activity questionnaires (PAQ) [54, 55] were applied. The validated 25-item FFQ [54, 55] was applied to calculate the dietary nutrient intakes (e.g. total energy intake (kcal/day), carbohydrate intake (g/day), protein intake (g/day), and fat intake (g/day)) based on the Chinese Food Composition Table (2004). The study was approved by the Human Research and Ethics Committee of the People’s Hospital of Nyingchi. The study was registered at ChiCTR.org.cn (ChiCTR1800016854) and was conducted in accordance with the principle of the Helsinki Declaration II. All the volunteers had given written informed consent.

**Figure 1.**
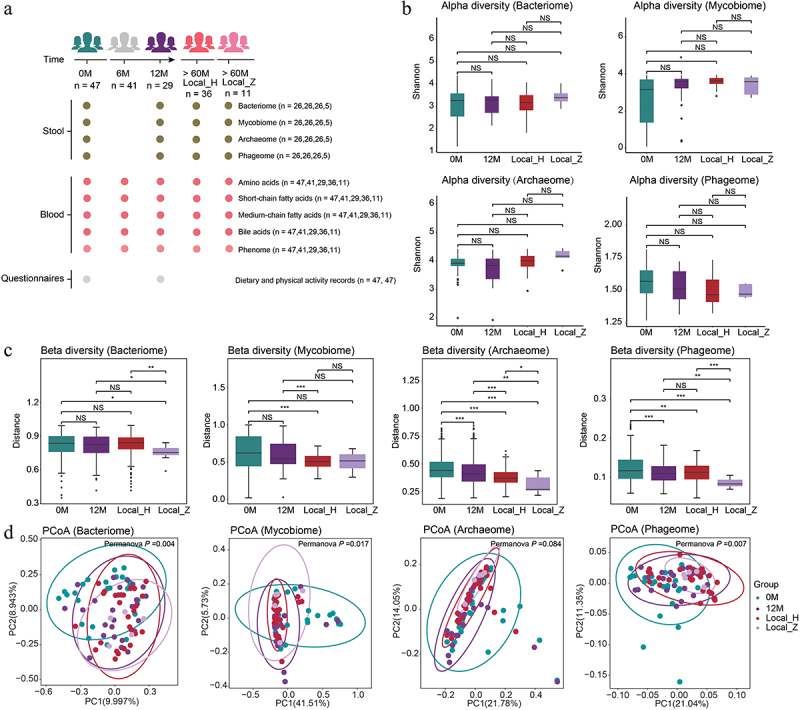
Schematic diagram of study design and alterations in the composition of gut multi-kingdom microorganisms in healthy individuals following moderate altitude exposure. (a) Schematic overview of the study design, depicting the total number of samples and participants from whom data were obtained. (b) Shannon index for alpha diversity at the species level. Plotted are interquartile ranges (IQRs; boxes), medians (dark lines in the boxes), the lowest and highest values within 1.5 times IQR from the first and third quartiles (lines above and below the boxes), and outliers beyond the lines (circles). (c) Beta-diversity for all samples at species level. Box plot as in B. (d) Principal coordinates analysis (PCoA) for individuals with moderate altitude exposure based on the gut microbiome using Bray–Curtis distance at the species level. Permutation multivariate analysis of variance (PERMANOVA) *P*-values of gut microbiome between individuals before and after moderate altitude exposure were shown. In figures b, c, and d, *n* = 26, 26, 26, and 5 for group 0 M, group 12 M, group Local_H, and group Local_Z, respectively. In figures b and c, *P*-value of paired/unpaired samples was calculated with paired/unpaired two-tailed Wilcoxon test. *p < 0.05; **p < 0.01; ***p < 0.001; NS, not significant.

After overnight fasting for at least 10 h, blood samples were collected from volunteers for clinical chemistry analyses. Serum samples were obtained through centrifugation and stored at −80°C for further analysis. Fecal samples were immediately snap-frozen in liquid nitrogen and stored at −80 °C for metagenome sequencing analysis. A Mindray BS600 analyzer was applied for routine blood tests and the measurement of FBG, triglycerides, low-density lipoprotein cholesterol (LDL), high-density lipoprotein cholesterol (HDL), total cholesterol (CHOL), creatinine, and uric acid. An Atellica IM1200 analyzer was applied for serum insulin measurement by chemiluminescence immunoassay method. And according to the manufacturer’s instructions, leptin and adiponectin were detected with ELISA kits (Elabsicence.cn and Guangdong Uniten Biotechnology Co., Ltd., respectively). In total, we performed metagenomics sequencing in 83 fecal samples and detected AAs, FAs (SCFAs, MCFAs) and BAs concentrations in 164 serum samples, respectively (Figure 1(a)).

### DNA extraction and sequencing

The fecal samples used for metagenomic sequencing were extracted using the sodium dodecyl sulphate method. All qualified DNA samples were applied for the library preparation and then sequenced with Illumina NovaSeq 6000 (Illumina, San Diego, USA) in PE150 mode. Details are described in the supplementary methods. A total of 871.1 Gb high-quality data (an average of 10.5 Gb/sample) were acquired after removing adaptor, low-quality reads and human DNA reads.

### Microbial taxonomic and functional profiles

#### Bacteria taxonomic profiling

Taxonomic profiling of the metagenomic samples was performed using metagenomic phylogenetic analysis 4 (MetaPhlAn 4, v4.0.6) [56], which used clade-specific markers to provide pan-microbial (i.e. bacterial, archaeal and eukaryotic) quantification at species-level. MetaPhlAn was run with parameters “–read_min_len 50 –ignore_eukaryotes – ignore_archaea.”

#### Fungi and archaea taxonomic profiling

The quality filtered reads were taxonomically classified using Kraken 2 (v2.1.3) [57] using the RefSeq database (fungi or archaea) as a reference with default parameters. The species abundance estimation was improved using Bracken (v2.6.2) [58]. The counts were normalized to 100%.

#### Bacteriophages taxonomic profiling

The reads in each sample were assembled with SPAdes assembler (v3.15) [59] in metagenomic mode. The contigs with length <1 kb were discarded. The contigs constructed from the viral fraction were screened with a gene enrichment-based method VirSorter 2 (v2.2) [60] and a k-mer frequency-based method VirFinder (v1.1) [61] to identify and remove bacteria-like contigs. CD-HIT (v.4.8) [62] was used to cluster pooled contigs (non-redundant contigs) at 95% global average nucleotide identity (−c 0.95). We classified viral contigs using viral NCBI RefSeq genomes and protein databases. To improve the viral protein homology search classification of viral contigs, we additionally used the phage structural proteins in the PfamA annotation with an *E* value < 1e-10. Finally, family level taxonomic annotations were assigned to the contigs that were not classified in the previous step using Demovir script with default parameters and database [63]. In bacteriophages-bacterial host interaction analysis, we used clustered regularly interspaced short palindromic repeat (CRISPR) Recognition Tool (v1.6.0) [64] to predict the CRISPR spacer sequences of bacterial genomes (Refseq reference genomes) and aligned the CRISPR spacer sequences with bacteriophage contigs. The unaligned contigs were further aligned with the microbe versus phage (MVP) database [65] to predict the possible bacterial hosts of the bacteriophage contigs. Finally, we combined the prediction results of the two methods.

#### Microbial functional profiles

Gene abundance profile was calculated as previously described [66]. Functional profiling was performed with the Human Microbiome Project Unified Metabolic Analysis Network 3 (HUMAnN 3) (v3.6.1) pipeline [67] using the search mode of UniProt Reference Clusters (uniref90) and pathway MetaCyc, which contained the functional potential of each metagenome sample. Details are described in the supplementary methods.

### Metagenomic sequencing data analysis

Only species present in >10% of samples were used for further analysis. Alpha diversity (i.e. Shannon index) was calculated to analyze the complexity of species diversity. Beta diversity was calculated to evaluate the differences in species complexity among samples. Principal coordinate analysis (PCoA) (i.e. Bray-Curtis distance) was used to visualize the difference in gut microbiome composition with vegan and ggplot2. Permutation multivariate analysis of variance (PERMANOVA) (Bray-Curtis distance and 999 permutation times) was used to evaluate the impacts of moderate altitude exposure on microbial profiles via vegan in R.

### Targeted circulating metabolites measurements

#### AAs profiling

AAs concentrations were detected by ultra-high performance liquid chromatography – mass spectrometer (UHPLC-MS/MS) as previously described with modifications [68]. Details are described in the supplementary methods.

#### FAs (including SCFAs and MCFAs) profiling

FAs concentrations were measured by gas chromatography – mass spectrometer (GC-MS) as previously described with modifications [69]. Details are described in the supplementary methods.

#### BAs profiling

BAs concentrations were assessed by UHPLC-MS/MS according to the method described previously with modifications [70]. Details are described in the supplementary methods.

### Statistical analysis

Serum metabolome (i.e. AAs, FAs, BAs) profiling was analyzed by Principal Component Analysis (PCA) with SIMCA 16 (Umeå, Sweden). All statistical analyses were made in the R software (v4.3.3). Categorical data were compared with Fisher’s exact test. *P*-value of paired or unpaired samples was calculated with paired or unpaired two-tailed Wilcoxon test, respectively. *P*-value for discontinuous variables was calculated by chi-square tests. *P*-values were adjusted by the Benjamini–Hochberg correction for multiple tests when required. Unless otherwise stated, *P*-value after false discovery rate correction <0.05 was considered as statistically significant level. Linear mixed model (LME) was conducted to assess the clinical index alterations over moderate altitude exposure time using the “lme4” package (v1.1). We first linearly transformed each clinical indices and standardized the total variation to 1 before applying lmer() function from the “lme4” R package, with the following formula as: lmer(Exp ~1 + exposure time + (1|SubjectID), data = data set, REML = FALSE), where Exp represented the linearly transformed and standardized values of each clinical index. Intra-kingdom or inter-kingdom correlations among bacteriome, mycobiome, archaeome and phageome were analyzed by Spearman’s rank correlation method. Multivariate predictive modelling of each omics dataset was conducted using partial least square-discriminant analysis incorporated into a repeated double cross-validation framework (rdcv-PLS) with “mixOmics” (v6.8.5) [71] with defaults. The effect size of FBG in impacting gut microbiota and serum metabolome variation were analyzed by PERMANOVA (Bray – Curtis distance). The association between each important variable and lowered FBG benefits was estimated by multivariate logistic regression analysis with glm() function from the “stats” R package (v4.3.3).

## Results

### Moderate altitude exposure affected the phenome of the study participants

The impacts of moderate altitude exposure on the phenome of the study individuals were shown in Table S1. Among the 19 clinical indices examined (excluding age, gender, and height), two clinical indices (DBP and HR) showed a significant increase, while nine indices (BMI, BW, FBG, insulin, serum creatinine, leptin, LDL, CHOL, and HDL) showed a significant decrease at the end of the study in contrast with baseline levels. Among them, the two increased clinical indices (DBP and HR) and the six decreased clinical indices (FBG, insulin, serum creatinine, leptin, CHOL, and HDL) were remarkably altered in relation to the duration of moderate altitude exposure revealed by the LME model (adjusted *p* < 0.05; Table S2). Notably, all participants remained healthy, as these clinical indices fluctuated within their normal ranges. Furthermore, the results of FFQ indicated no significant changes in dietary nutrient intake (*p* > 0.05; Table S3) in individuals before and after moderate altitude exposure. It was well-known that intensive physical activity might contribute to the improvements in metabolic health and reductions in blood glucose. However, PAQ showed that individuals exhibited reduced intensity and frequency of physical activities following moderate altitude exposure (*p* < 0.05; Table S3). These findings indicated that the observed alterations in clinical indices (e.g. BW, BMI, and FBG) were more likely to be influenced by moderate altitude exposure rather than by dietary changes or physical activity modifications.

### Association between alterations in gut bacteria composition and moderate altitude exposure

**Figure 2.**
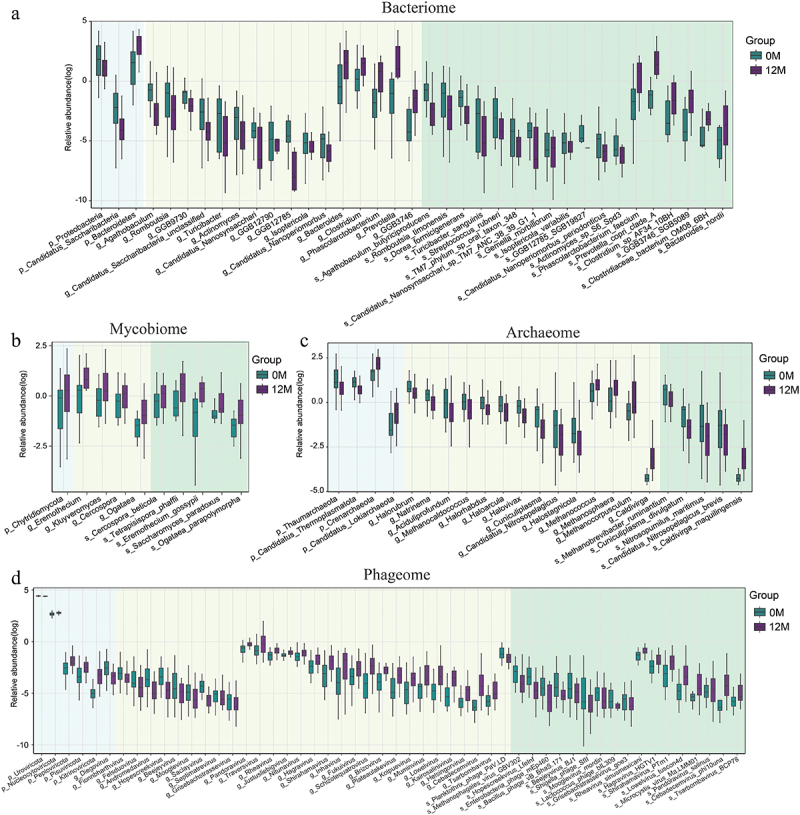
Alterations in gut multi-kingdom microorganisms in healthy individuals following moderate altitude exposure. (a-d) relative abundance of differential bacteria, fungi, archaea, and bacteriophages in individuals with moderate altitude exposure at the phylum, genus, and species levels (adjusted *p* < 0.1). *n* = 26 and 26 for group 0 M and group 12 M, respectively. In figures a, b, c, and d, boxplot plot as in Figure 1b, and *p*-value was calculated with paired two-tailed Wilcoxon test after false discovery rate correction.

Shotgun metagenomic sequencing was applied to investigate the alterations in the gut bacteria of individuals before and after moderate altitude exposure (Figure 1(a); Table S4). No significant difference in bacterial alpha diversity was observed in individuals before and after moderate altitude exposure over 12 months (group 12 M) (Figure 1(b)). The 12 M group showed significantly lower bacterial beta diversity at the genus level; however, no difference was noted at the phylum and species levels when compared with baseline (Figure 1(c); Figure S1A-S1B). PCoA revealed significant changes in the gut bacterial composition of individuals before and after moderate altitude exposure at the phylum, genus, and species levels (PERMANOVA *p* = 0.006, 0.001, and 0.004, respectively) (Figure 1(d); Figure S1C-S1D). At the phylum level, the relative abundance of two phyla (*Candidatus Saccharibacteria* and *Proteobacteria*) decreased and that of one phylum (*Bacteroidetes*) increased in the 12 M group (adjusted *p* < 0.05; Figure 2(a); Table S5). At the genus level, the relative abundance of five genera (*Agathobaculum*, *Romboutsia*, *Isoptericola*, *Turicibacter* and *Candidatus Nanosynsacchari*) decreased and that of three genera (*Prevotella*, *GGB3746*, and *Phascolarctobacterium*) increased in the 12 M group (adjusted *p* < 0.05; Figure 2(a); Table S5). At the species level, the relative abundance of four species (*Agathobaculum butyriciproducens*, *Romboutsia timonensis*, *TM7_phylum_sp_oral_taxon_348*, and *Isoptericola variabilis*) decreased in the 12 M group (adjusted *p* < 0.05; Figure 2(a); Table S5). Furthermore, no differences in bacterial alpha diversity were observed between the healthy Han Chinese population residing in Nyingchi (>5 years) (group Local_H) and healthy Tibetans population (group Local_Z) residing in Nyingchi (>5 years) (Figure 1(b)). Bacterial beta diversity at the phylum, genus and species levels was consistently lower in the Local_Z group than in the Han Chinese group, regardless of residence time (Figure 1(c); Figure S1A-S1B). PCoA indicated that the gut bacterial composition of individuals exposed to moderate altitude gradually converged towards that of long-term Nyingchi residents (>5 years) (Figure 1(d); Figure S1C–S1D).

### Association between alterations in gut fungi composition and moderate altitude exposure

Next, we evaluated alterations in gut fungi composition associated with moderate altitude exposure. The 12 M group showed a slight increase in fungal alpha diversity at 12 months of exposure as compared to that at baseline (Figure 1(b)). We observed notably lower fungal beta diversity at the genus level; however, no difference was found at the phylum and species levels in the 12 M group when compared with baseline (Figure 1(c); Figure S1A-S1B). PCoA revealed significant changes in the gut fungi composition of individuals before and after moderate altitude exposure at the genus and species levels, but not at the phylum level (PERMANOVA *p* = 0.023, 0.017, and 0.424, respectively) (Figure 1(d); Figure S1C-S1D). At the phylum level, the relative abundance of one phylum (*Chytridiomycota*) increased in the 12 M group (adjusted *p* < 0.1; Figure 2(b); Table S6). At the genus level, the relative abundance of four genera (*Cercospora*, *Kluyveromyces*, *Ogataea*, and *Eremothecium*) increased in the 12 M group (adjusted *p* < 0.1; Figure 2(b); Table S6). At the species level, the relative abundance of five species (*Cercospora beticola*, *Tetrapisispora phaffii*, *Ogataea parapolymorpha*, *Eremothecium gossypii*, and *Saccharomyces paradoxus*) increased in the 12 M group (adjusted *p* < 0.1; Figure 2(b); Table S6). Besides, the Local_H group showed a significant increase in fungal alpha diversity as compared to the 0 M group (Figure 1(b)). The Local_H group exhibited significantly lower fungal beta diversity at the phylum, genus and species levels than the 12 M group (Figure 1(c); Figure S1A-S1B). PCoA suggested that the gut fungi composition of individuals exposed to moderate altitude became analogous to that of long-term residents of Nyingchi (>5 years) (Figure 1(d); Figure S1C–S1D).

### Association between alterations in gut archaea composition and moderate altitude exposure

Furthermore, alterations in gut archaea composition associated with moderate altitude exposure were investigated. No significant difference in archaeal alpha diversity was observed in the 12 M group when compared with that at baseline (Figure 1(b)). The 12 M group exhibited significantly higher archaeal beta diversity at the phylum level but lower archaeal beta diversity at the genus and species levels when compared with baseline results (Figure 1(c); Figure S1A-S1B). PCoA suggested that significant changes in the gut archaea composition of individuals before and after moderate altitude exposure at the phylum level but not at the genus and species levels (PERMANOVA *p* = 0.002, 0.068, and 0.084, respectively) (Figure 1(d); Figure S1C-S1D). At the phylum level, the relative abundance of two phyla (*Thaumarchaeota* and *Candidatus Thermoplasmatota*) decreased, while the relative abundance of two phyla (*Crenarchaeota* and *Candidatus Lokiarchaeota*) increased in the 12 M group (adjusted *p* < 0.05; Figure 2(c); Table S7). At genus level, the relative abundance of three genera (*Cuniculiplasma*, *Candidatus Nitrosopelagicus*, and *Haloarcula*) decreased, while the relative abundance of one genus (*Caldivirga*) increased in the 12 M group (adjusted *p* < 0.05; Figure 2(c); Table S7). At the species level, the relative abundance of four species (*Cuniculiplasma divulgatum*, *Candidatus Nitrosopelagicus brevis*, *Methanobrevibacter ruminantium*, and *Nitrosopumilus maritimus*) decreased and that of one species (*Caldivirga maquilingensis*) increased in the 12 M group (adjusted *p* < 0.1; Figure 2(c); Table S7). Additionally, the Local_H group and Local_Z group showed no differences in archaeal alpha diversity between them but exhibited an increasing trend following long-term residence (Figure 1(b)). Significantly lower archaeal beta diversity at phylum, genus, and species levels was identified in the Local_H group and Local_Z group compared to that in the 12 M group, with the Local_Z group showing the lowest archaeal beta diversity (Figure 1(c); Figure S1A-S1B). PCoA showed that the gut archaea composition of individuals exposed to moderate altitude gradually converged towards that of long-term residents in Nyingchi (>5 years) (Figure 1(d); Figure S1C–S1D).

### Association between alterations in gut bacteriophage composition and moderate altitude exposure

Moreover, we assessed the association between alterations in gut bacteriophage composition and exposure to moderate altitude. The 12 M group showed no significant changes in bacteriophagic alpha diversity when compared with that at baseline (Figure 1(b)). Significantly lower bacteriophagic beta diversity was observed at the phylum and species levels in the 12 M group as compared to that at baseline, while no difference was noted at the genus level (Figure 1(c); Figure S1A-S1B). PCoA revealed distinct clustering patterns among the samples from individuals before and after moderate altitude exposure at the phylum, genus and species levels (PERMANOVA *p* = 0.004, 0.003, 0.007, respectively) (Figure 1(d); Figure S1C-S1D). At the phylum level, the relative abundance of one phylum (*Uroviricota*) decreased and that of two phyla (*Nucleocytoviricota* and *Kitrinoviricota*) increased in the 12 M group (adjusted *p* < 0.05; Figure 2(d); Table S8). At genus level, the relative abundance of one genus (*Beejeyvirus*) decreased, while that of 10 genera (*Pandoravirus*, *Schizotequatrovirus*, *Brizovirus*, *Cebadecemvirus*, *Tsarbombavirus*, *Kairosalinivirus*, *Inhavirus*, *Rheavirus*, *Helsingorvirus*, and *Shirahamavirus*) increased in the 12 M group (adjusted *p* < 0.05; Figure 2(d); Table S8). At the species level, the relative abundance of 10 species (*Lactococcus phage bIL309*, *Beejeyvirus BJ1*, *Shigella phage SfII*, *Enterobacteria phage mEp460*, *Methanophagales virus GBV302*, *Mooglevirus mordin*, *Grisebachstrassevirus goe3*, *Hopescreekvirus LfeInf*, *Planktothrix phage PaV-LD*, and *Bacillus phage vB_BhaS-171*) decreased, while the relative abundance of eight species (*Cebadecemvirus phi10una*, *Rheavirus sinusmexicani*, *Shirahamavirus PTm1*, *Tsarbombavirus BCP78*, *Lowelvirus tuscon4d*, *Pandoravirus salinus*, *Hagravirus HGTV1*, and *Microcystis virus Ma-LMM01*) increased in the 12 M group (adjusted *p* < 0.1; Figure 2(d); Table S8). Besides, the Local_H group and Local_Z group showed no differences in bacteriophagic alpha diversity among them but exhibited a decreasing trend following long-term residence (Figure 1(b)). Compared to the 12 M group, the Local_H group showed significantly higher bacteriophagic beta diversity at the phylum and genus levels but not at the species level, while the Local_Z group showed significantly lower bacteriophagic beta diversity at the genus and species levels but not at the phylum level (Figure 1(c); Figure S1A-S1B). The Local_Z group showed lower bacteriophagic beta diversity at the phylum, genus and species levels than the Local_H group. PCoA suggested that the gut bacteriophage composition of individuals exposed to moderate altitude became analogous to that of long-term residents (>5 years) in Nyingchi (Figure 1(d); Figure S1C-S1D).

### Functional characterization of the gut microbiome associated with moderate altitude exposure

For functional annotation, we used the HUMAnN 3 pipeline to investigate which genes engaged MetaCyc pathways were associated with moderate altitude exposure. Among 475 PWY pathways, 15 PWY pathways were significantly elevated in the microbiome of the 12 M group (adjusted *p* < 0.05; Additional File 1: Figure S2A-B and Table S9). The top five significantly upregulated pathways were DTDPRHAMSYN-PWY (dTDP-&beta;-L-rhamnose biosynthesis), PWY-6807 (xyloglucan degradation II (exoglucanase)), PANTOSYN-PWY (superpathway of coenzyme A biosynthesis I (bacteria)), PYRIDNUCSYN-PWY (NAD de novo biosynthesis I (from aspartate)), and PANTO-PWY (phosphopantothenate biosynthesis I)). These pathways were predominantly driven by *Bacteroidetes* species (e.g. *Bacteroides vulgatus*, *Bacteroides uniformis*, and *Bacteroides plebeius*) and SCFAs producers (e.g. *Faecalibacterium prausnitzii*, *Roseburia faecis*, *Eubacterium rectale*, and *Megamonas funiformis*). Conversely, 13 PWY pathways were significantly downregulated in the microbiome of the 12 M group (adjusted *p* < 0.05; Figure S2A, S2C and Table S9). The top five significantly downregulated pathways were PPGPPMET-PWY (ppGpp metabolism), PWY-6612 (superpathway of tetrahydrofolate biosynthesis), FOLSYN-PWY (superpathway of tetrahydrofolate biosynthesis and salvage), LIPA-CORESYN-PWY (Lipid A-core biosynthesis), and PWY-5367 (petroselinate biosynthesis)). These pathways were predominantly driven by *Proteobacteria* species (e.g. *Escherichia coli* and *Klebsiella pneumoniae*). PCoA revealed that the functions of the microbiome of individuals exposed to moderate altitude became similar to those of long-term residents (>5 years) in Nyingchi (Figure S2D).

### Alterations in the multi-kingdom correlation network in individuals exposed to moderate altitude exposure

**Figure 3.**
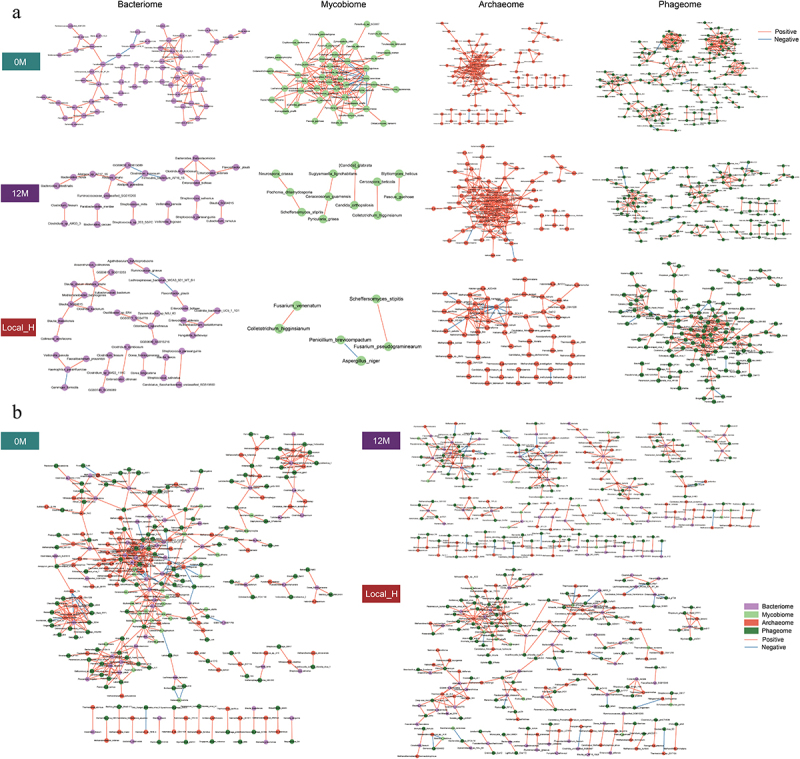
Correlation analysis results of gut multi-kingdom microorganisms in healthy individuals following moderate altitude exposure. (a) The intra-kingdom associations among all gut bacteria, fungi, archaea, and bacteriophages at species level by Spearman’s rank correlation analysis in group 0 M (*n* = 26), group 12 M (*n* = 26) and group Local_H (*n* = 26), respectively. (b) The inter-kingdom associations among all gut multi-kingdom microorganisms at species level by Spearman’s rank correlation analysis in group 0 M, group 12 M and group Local_H, respectively. Purple dots, bacteria; light green dots, fungi; brick red dots, archaea; dark green dots, bacteriophages. Red edges, Spearman’s rank correlation coefficient > 0.7, adjusted *p* < 0.05; blue edges, Spearman’s rank correlation coefficient < −0.7, adjusted *p* < 0.05.

To elucidate the potential interactions between multi-kingdom species and their role in healthy Han Chinese individuals following moderate altitude exposure, we performed Spearman’s rank correlation analysis based on the abundance of species present in > 50% of samples. Generally, the intra-kingdom correlation network of individuals before moderate altitude exposure (340 species and 649 associations) was more complex than that of individuals after moderate altitude exposure for 12 months (229 species and 402 associations) or more than 5 years (212 species and 303 associations) (Spearman’s |*rho*| > 0.7 and adjusted *p* < 0.05; Figure 3(a); Table S10-S12). The correlations networks in archaea and bacteriophages were more intensive than those of bacteria and fungi, independent of moderate altitude exposure. In addition to intensive correlations between intra-kingdom species, we observed substantial associations between inter-kingdom species of individuals before moderate altitude exposure (286 species and 449 associations), which were more complex than those of individuals after moderate altitude exposure for 12 months (239 species and 219 associations) or more than 5 years (207 species and 218 associations) (Spearman’s |*rho*| > 0.7 and adjusted *p* < 0.05; Figure 3(b); Table S13-S15). Notably, more significant correlations were observed between the bacteria and the bacteriophages. Otherwise, there were considerably more positive correlations than negative correlations observed in both intra-kingdom and inter-kingdom species correlations. Taken together, these findings suggested an important role of the interactions between intra-kingdom and inter-kingdom microorganisms in individuals following moderate altitude exposure.

### Viral host detection based on the CRISPR spacer sequences and MVP database

**Figure 4.**
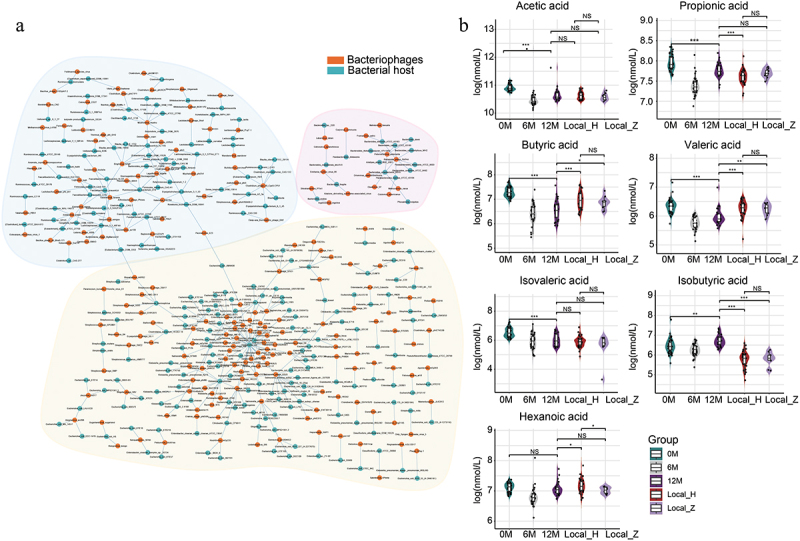
Bacteriophages – bacterium interactions analysis and alterations in circulating short-chain fatty acids (SCFAs) in individuals following moderate altitude exposure. (a) Taxonomically annotated virus–host interactions based on the clustered regularly interspaced short palindromic repeat (CRISPR) spacer sequences and the microbe versus phage (MVP) database at the species level of bacteriophages. (b) The serum concentrations of SCFAs in healthy individuals following moderate altitude exposure. *n* = 47, 41, 29, 36, and 11 for group 0 M, group 6 M, group 12 M, group Local_H, and group Local_Z, respectively. Plotted are interquartile ranges (IQRs; boxes), medians (dark lines in the boxes), the lowest and highest values within 1.5 times IQR from the first and third quartiles (lines above and below the boxes), and density of values (width between curves). *P*-value of paired/unpaired samples was calculated with paired/unpaired two-tailed Wilcoxon test. *p < 0.05; **p < 0.01; ***p < 0.001; NS, not significant. Light red, most virus–host interactions were bacteriophages-*Bacteroidetes*; light yellow, most virus–host interactions were bacteriophages-*Proteobacteria*; light cyan, most virus–host interactions were bacteriophages-SCFAs producers.

To further elucidate the bacteriophage-bacterium interaction, we analyzed the CRISPR loci of bacterial contigs from all samples and combined MVP database alignment results to identify infected viruses. We identified 129, 448, and 579 unique taxonomically annotated virus–host interactions at the bacteriophage family, genus, and species levels, respectively (Figure 4(a); Figure S3A–S3B and Table S16). Generally, the virus–host interactions could be classified into three categories: bacteriophages-*Bacteroidetes* (e.g. *Bacteroides ovatus*, *Bacteroides uniformis*, *Bacteroides cellulosilyticus*, *Bacteroides thetaiotaomicron*, and *Parabacteroides distasonis*), bacteriophages-*Proteobacteria* (e.g. *Escherichia coli*), and bacteriophages-SCFAs producers (e.g. *Butyricicoccus*, *Ruminococcus*, *Faecalibacterium*, *Blautia*, *Lachnospiraceae*, *Roseburia*, *Clostridium*, and *Eubacterium*). These data indicated potential changes in SCFAs in individuals following moderate altitude exposure. Subsequently, we measured seven SCFAs in all serum samples. In contrast with baseline, the levels of five SCFAs (acetic acid, propionic acid, butyric acid, valeric acid, and isovaleric acid) decreased, the level of one SCFA (isobutyric acid) increased, and the level of one SCFA (hexanoic acid) remained unchanged in the 12 M group (Figure 4(b)). With prolonged exposure time, the butyric acid and valeric acid levels increased, while the isobutyric acid level decreased. These results demonstrated that the changes in the composition, functions, and interactions of gut multi-kingdom microorganisms were associated with the alterations in serum SCFAs in individuals following moderate altitude exposure.

### Association analysis among bacteria-fungi-archaea-bacteriophages-metabolites-clinical phenotypes

**Figure 5.**
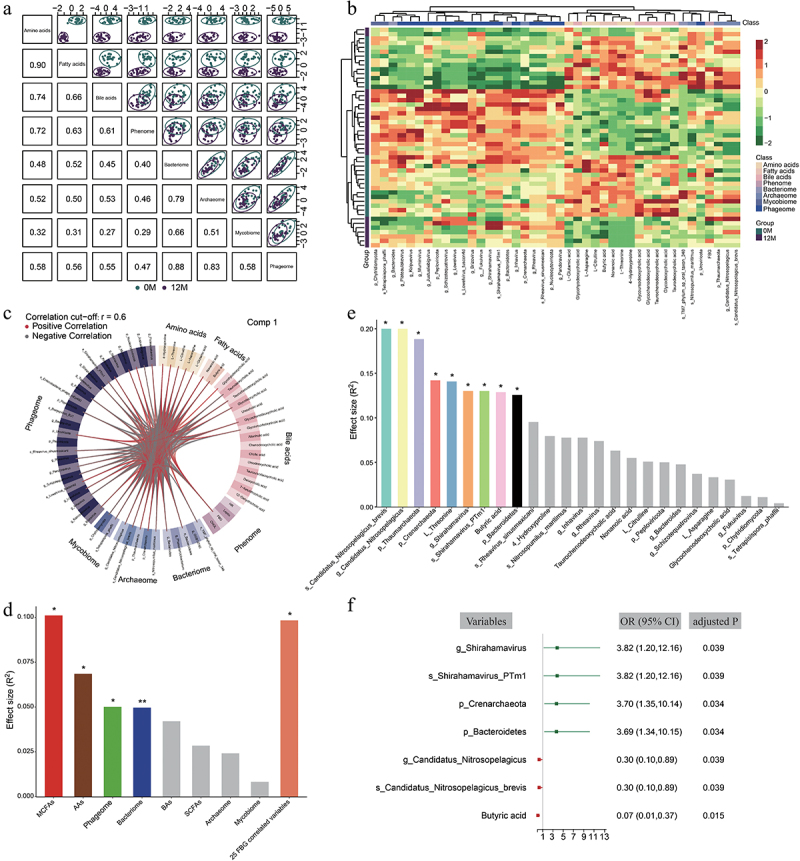
Model performance of the integrative modeling of bacteria-fungi-archaea-bacteriophages-metabolome-clinical phenome interactions in healthy individuals (*n* = 26) before and after moderate altitude exposure. (a) The model performance of DIABLO integrative modeling on OMICs signatures. (b) a clustered image map (Euclidean distance, complete linkage) of the multi-OMICs signature. Samples are represented in rows, and selected features on the first component are represented in columns. (c) The Circos plot shows the positive (negative) correlations, denoted as brown (grey) lines, between selected multiOMIC features. (d) The effect size of fasting blood glucose (FBG) on variations in the gut microbial profiles, serum metabolome profiles, and selected FBG associated multiOMIC features. Covariates were identified via envfit (vegan) and those with statistical significance were colored and marked. *PERMANOVA *p* < 0.05; **PERMANOVA *p* < 0.01. (e) The effect size of each selected FBG associated multiOMIC feature on the FBG variation. Covariates were identified via PERMANOVA and those with statistical significance were colored and marked. *PERMANOVA adjusted *p* < 0.05. (f) The or per standard deviation increment and 95% CI estimation for the association between 7 FBG associated multiOMIC features and increased lower FBG benefits by multi-logistic regression analysis (adjusted *p* < 0.05). Green lines, positive association; red lines, negative association.

To illustrate the potential systemic and physiological interactions among the gut microbiota, serum metabolites, and clinical phenotypes in healthy individuals exposed to moderate altitude, we constructed biological networks of gut microbiota (37 bacteria, 10 fungi, 23 archaea, and 52 bacteriophages, adjusted *p* < 0.1 at the phylum, genus and species levels), targeted serum metabolites (24 AAs, 11 FAs (7 SCFAs and 4 MCFAs), and 24 BAs), and 16 clinical indices. The gut microbiota relevant metabolites and clinical indices were investigated using the rdcv-PLS model. Different groups could be discriminated on the first component of the model for the serum metabolome (particularly datasets for AAs and FAs), clinical phenome, and gut microbiota datasets (Figure 5(a)). Besides, we observed strong correlations between AAs and other datasets as well as among bacteria, archaea and bacteriophage datasets. However, the fungal datasets showed weak correlations with the serum metabolome and clinical phenome and modest correlations with other microbiota. In total, rdcv-PLS model contributed to the screening of 63 vital variables (Figure 5(b)). Then, on the condition of |Pearson’s correlation coefficients| > 0.6, rdcv-PLS analysis selected 42 vital variables (3 bacteria, 2 fungi, 5 archaea, 18 bacteriophages, 5 AAs, 2 FAs, 6 BAs, and 1 clinical index) (Figure 5(c); Table S17). Particularly, FBG was the sole clinical index selected by the rdcv-PLS model. Next, we focused on the associations between vital variables and FBG, and found that 25 vital variables were significantly associated with FBG. Specifically, 12 variables showed a positive correlation with FBG, including 4 archaea (p_*Thaumarchaeota*, g_*Candidatus_Nitrosopelagicus*, s_*Nitrosopumilus_maritimus*, and s_*Candidatus_Nitrosopelagicus_brevis*), 4 AAs (L-Threonine, 4-Hydroxyproline, L-Asparagine, and L-Citrulline), 2 FAs (Butyric acid and Nonanoic acid) and 2 BAs (Glycochenodeoxycholic acid and Taurochenodeoxycholic acid). Conversely, 13 variables showed a negative correlation with FBG, including 2 bacteria (p_*Bacteroidetes* and g_*Bacteroides*), 2 fungi (p_*Chytridiomycota* and s_*Tetrapisispora_phaffii*), 1 archaea (p_*Crenarchaeota*) and 8 bacteriophages (p_*Peploviricota*, g_*Fukuivirus*, g_*Inhavirus*, g_*Rheavirus*, g_*Schizotequatrovirus*, g_*Shirahamavirus*, s_*Rheavirus_sinusmexicani*, and s_*Shirahamavirus_PTm1*) (Figure 5(c); Figure S4).

The interactions between the FBG level and the multi-kingdom microorganisms’ profiles and targeted serum profiles were further evaluated by PERMANOVA. Overall, the FBG level showed significant impacts on the MCFAs, AAs, phageome, and bacteriome profiles, with effect sizes (R^2^) ranging from 0.10 to 0.04 in descending order (PERMANOVA *p* < 0.05; Figure 5(d); Table S18). The FBG level exhibited an explanatory power of 9.83% on the variations of the aforementioned 25 vital variables. Moreover, the influence of 25 FBG-correlated variables on the FBG level was evaluated by PERMANOVA. Totally, nine variables (s_*Candidatus_Nitrosopelagicus_brevis*, g_*Candidatus_Nitrosopelagicus*, p_*Thaumarchaeota*, p_*Crenarchaeota*, L-Threonine, g_*Shirahamavirus*, s_*Shirahamavirus_PTm1*, butyric acid, and p_*Bacteroidetes*) showed significant influence on the FBG, with effect sizes ranging from 0.19 to 0.12 in descending order (PERMANOVA adjusted *p* < 0.05; Figure 5(e); Table S19).

Furthermore, the associations between the above-mentioned nine vital variables and lowered FBG benefits in individuals exposed to moderate altitude (*n* = 26) were assessed using multivariate logistic regression analysis. We found that four and three variables were positively and negatively associated with lowered FBG benefits, respectively (adjusted *p* < 0.05; Figure 5(f); Table S20). Among these, s_*Shirahamavirus_PTm1* (OR = 3.82; 95% CI: 1.20–12.16), p_*Crenarchaeota* (OR = 3.70; 95% CI: 1.35–10.14), and p_*Bacteroidetes* (OR = 3.69; 95% CI: 1.34–10.15) might be helpful for FBG intervention even improving the efficacy of diabetes prevention. Conversely, s_*Candidatus_Nitrosopelagicus_brevis* (OR = 0.30; 95% CI: 0.10–0.89) and butyric acid (OR = 0.07; 95% CI: 0.01–0.37) might be complementary to glucose in improving dysglycemia or diabetes forewarning, which should be further validated in the future study. These findings suggested that the interactions between bacteria, fungi, archaea, bacteriophages and serum metabolites may influence the lowered FBG in individuals exposed to moderate altitude.

## Discussion

Previous research had demonstrated that high-altitude exposure influencing host phenotype correlated with alterations in gut bacteria in humans and other animals, such as mice and yaks [40, 47–52]. However, there was limited understanding of how long-term (~12 months) moderate altitude exposure modulated the multi-kingdom microorganisms (bacteria, fungi, archaea, and bacteriophages) within the gut ecosystem of healthy individuals. This study delineated the landscapes and interaction networks of multi-kingdom microorganisms in the gut ecosystem, serum metabolites, and phenome in the individuals exposed to moderate altitude. The composition, functions and correlations of gut multi-kingdom microorganisms differed in individuals following moderate altitude exposure. Moreover, we identified significant associations between multi-kingdom species and serum metabolites with FBG in individuals exposed to moderate altitude.

Previous research had shown that *Proteobacteria* was positively associated with MetS, which was contrary to *Bacteroidetes* [16, 72, 73]. Experimental studies in mice had shown that gavage with *Bacteroidetes* species (e.g. *Bacteroides thetaiotaomicron* or *Bacteroides ovatus*) could alleviate diet-induced body weight gain and adiposity [74–76]. Additionally, inhibiting the growth of *Proteobacteria* species (e.g. *Escherichia coli*) could attenuate hyperglycemia in db/db mice [77], which was consistent with our findings. The current study found that the upregulation of *Bacteroidetes* and the downregulation of *Proteobacteria* were associated with lower BMI and FBG in individuals exposed to moderate altitude. Besides, Increased *Phascolarctobacterium* was observed in individuals exposed to moderate altitude, which was observed to be negatively associated with obesity in the previous research [78–80]. Increased *Prevotella* potentiated weight loss and decreased cholesterol levels [81], while decreased *Romboutsia timonensis* showed an association with a lower LDL-cholesterol level [82], which was in accordance with reduced BW, cholesterol and LDL in individuals exposed to moderate altitude.

MetaPhlAn 4 was a method of integrating information from metagenome assemblies and genomes of microbial isolates to expand metagenomic taxonomic profiling. As we had previously investigated the metagenome data with MetaPhlAn 2, a previous version for metagenomic taxonomic profiling, we compared the results of these two versions. Overall, the results of alterations in bacterial alpha diversity and beta diversity as well as taxonomic alterations were similar in these two versions. These observations confirmed that the gut bacterial composition varied in individuals before and after moderate altitude exposure, which were characterized with decreased *Proteobacteria* (e.g. *Escherichia coli*) and increased *Bacteroidetes* (e.g. *Bacteroides ovatus*, *Bacteroides thetaiotaomicron*, *Bacteroides uniformis*, *Bacteroides cellulosilyticus*, *Bacteroides nordii*, and *Parabacteroides distasonis*). Additionally, MetaPhlAn 4 provided new insights into altered bacterial populations in individuals exposed to moderate altitude, such as unknown species with species-level genome bins (Figure 2(a); Table S5). It also identified known decreased species (*Agathobaculum butyriciproducens* and *Romboutsia timonensis*), offering new clues to better understand the potential mechanisms underlying the decrease in SCFAs, BW, and potentially harmful lipids, which deserved further research. Besides, among the top 10 significantly altered pathways revealed by HUMAnN 3, four pathways (downregulated PPGPPMET-PWY, PWY-6612, and FOLSYN-PWY, but upregulated PANTO-PWY) were also identified by HUMAnN 2. These concordances suggested that these four altered pathways and their associated metabolic changes were important for individuals exposed to moderate altitude.

In contrast with gut bacteria, fungi exhibited modest changes in individuals exposed to moderate altitude when compared with baseline results. The rdcv-PLS model revealed weak correlations between fungi datasets and serum metabolome and other microbiota datasets. We found plant pathogens (Cercospora beticola [83, 84], *Eremothecium gossypii* [85, 86]) and yeast (*Kluyveromyces* [87, 88], *Tetrapisispora phaffii* [89, 90], *Ogataea parapolymorpha* [91], *Saccharomyces paradoxus* [92, 93]) increased in individuals exposed to moderate altitude. These gut fungi possessed various environmental counterparts, indicating potential origins or sources of the fungi and indicating how human health might be affected by the surrounding environments.

In addition, ammonia-oxidizing archaea (e.g. *Thaumarchaeota* [18], *Candidatus Nitrosopelagicus* [94], *Candidatus Nitrosopelagicus brevis* [95, 96], *Nitrosopumilus maritimus* [97]) decreased, while pulmonary tuberculosis associated *Crenarchaeota* [98] increased in individuals with moderate altitude exposure. Interestingly, these five archaea were identified by the rdcv-PLS model as vital variables associated with FBG. However, the specific mechanisms through which these archaea affect FBG *in vitro and in vivo* require further investigations. Methanogenic archaea in the gut functioned in the fermentative digestion of dietary fibers, favoring the production of SCFAs [99]. We found *Methanobrevibacter ruminantium* [100, 101], a methane producer, decreased in individuals with moderate altitude exposure, indicating reduced SCFAs production, which aligned with the observed alterations in gut bacteria composition. Like fungi, a literature review suggested that the gut archaea identified in this study were originally discovered and isolated from extreme ecosystems, highlighting the complex interrelationships among environments, archaea, and human health.

Besides, we identified significantly altered bacteriophages in individuals exposed to moderate altitude, although there were limited literatures documenting their specific functions in human health. Further studies are clearly warranted to delineate how the specific bacteriophage affects human health and how they are in concert with other microbial species (e.g. bacteria, fungi or archaea) to modulate metabolism. Notably, *Kairosalinivirus* increased in individuals exposed to moderate altitude, which was reported to infect the *Bacteroidetes* with strictly lytic and wide host range [102]. Meanwhile, diabetic nephropathy associated *Shigella phage SfII* [103] and ulcerative colitis associated *Enterobacteria phage mEp460* [104] were decreased, infecting the *Proteobacteria*. Correspondingly, the *Bacteroidetes* increased but the *Proteobacteria* decreased in individuals exposed to moderate altitude. Moreover, both the host–virus interaction analysis and inter-kingdom correlation analysis revealed alterations in the networks between SCFA producers and other microbiota, resulting in decreased SCFAs in individuals with moderate altitude exposure.

Otherwise, we observed diminished correlations among inter-kingdom and intra-kingdom microorganisms in individuals exposed to moderate altitude. A recent study reported that the co-occurrence network among fungi became increasingly complex with the deterioration of glycemic control, which was inversely associated with insulin sensitivity [105]. Thus, we speculated the attenuated interactions among multi-kingdom microorganisms might be correlated with the lowered FBG benefits in individuals with moderate altitude exposure, warranting future investigations. Preceding studies reported that moderate altitude could bring beneficial effects to host glucose homeostasis [1,106]. In the association analysis of multi-kingdom microorganisms, serum metabolites and clinical indices, we astonishingly found that many bacteriophages were negatively correlated with FBG. Specifically, *Shirahamavirus PTm1* was significantly associated with lowered FBG benefits, and its host *Bacteroides ovatus* (*p* = 5.34E–03) increased in individuals with moderate altitude exposure. *Bacteroides ovatus* was reported to produce indoleacetic acid to fortify intestinal barrier function via activation of intestinal aryl hydrocarbon receptor to relieve insulin resistance [76]. Recent studies had reported that FVT by removal of bacteria from lean donors could alter fecal microbiota and shift the phenotype of obese mice into closer resemblance of lean mice, even normalizing blood glucose tolerance [30, 33]. Whether and how *Shirahamavirus PTm1* interacted with *Bacteroides ovatus* to play the roles in glucose regulation, and whether FVT from healthy individuals with moderate altitude exposure to individuals with obesity and T2D might be helpful for glucose homeostasis, deserved further research.

The current study presented several limitations that warranted acknowledgment. First, the study was observational, and associations were not proof of causation. The specific mechanisms of moderate altitude exposure and the environmental factors (e.g. hypoxia, low barometric pressure, low temperature and increased ultraviolet radiation) that primarily drove changes in gut multi-kingdom microorganisms and host health required further elucidation through animal studies. Second, while we observed more significant changes in gut bacteriome and phageome profiles as compared to mycobiome and archaeome profiles in individuals following moderate altitude exposure, the underlined mechanisms remain unclear. Third, our findings suggested that after 12 months of moderate altitude exposure, the composition and functions of multi-kingdom microorganisms as well as the serum metabolome profiles of Han Chinese gradually converged towards that of Tibetan residents (Figure 1(d); Figure S1C-S1D, Figure S2D and Figure S5). These indicated that moderate altitude exposure, rather than genetic factors, mainly drove these alterations. However, due to the limited sample size, further research with much more independent cohorts with larger sample size and longer duration was necessary to explore and validate the longitudinal and ethnic effects. Fourth, more frequent longitudinal profiling of routine activity and dietary diversity would be helpful to ameliorate the influence of recall bias and measurement errors. Fifth, our analysis of the enteric phageome (i.e. DNA virome) from whole-metagenome shotgun sequencing reads excluded RNA viruses; thus, the gut RNA virome changes need elaboration through metatranscriptomics. Additionally, we did not enrich viral-like particles (VLP) in feces before sequencing. However, the conventional VLP concentration followed by sequencing could also bias isolation of viral taxa. We anticipate that these findings will inspire future exploration and development of novel therapeutic strategies (e.g. plateau trips, FVT, FMT, phage therapy) for cardiometabolic disease management.

## Conclusions

In summary, our findings extend our insights into the interplay among the moderate altitude exposure, gut multi-kingdom microorganisms, serum metabolome and host phenome. The observed alterations in multi-kingdom microorganisms and their significant associations with serum metabolome and lowered FBG benefits in individuals exposed to moderate altitude suggest moderate altitude exposure, FMT, or FVT from donors with moderate altitude exposure may be possible methods for FBG intervention. These findings open novel avenues to counteract the pathogenesis of dysglycemia and related metabolic disorders.

## Supplementary Material

Supplemental Material

Figure S3.tif

Figure S5.tif

Figure S1.tif

Figure S4.tif

Figure S2.tif

## Data Availability

Metagenomics sequencing data supporting the findings of this study were deposited in the European Nucleotide Archive (https://www.ebi.ac.uk/ena/browser/home; accession codes: PRJNA812695). Metabolomics data were deposited in the OMIX, China National Center for Bioinformation/Beijing Institute of Genomics, Chinese Academy of Sciences (https://ngdc.cncb.ac.cn/omix/releaseList; accession numbers: OMIX008071). The metagenome raw data, serum amino acid and propionic acid data, and phenome data were previously published (54). Data analysis in this study mainly relied on open-source tools, Trimmomatic, SOAP2, MetaPhlAn 4, Kraken 2, Bracken, SPAdes, VirSorter 2, VirFinder, CD-HIT, Demovir, CRISPR Recognition Tool and HUMAnN 3 for shotgun metagenomics sequencing. The aforementioned tools used for the data analysis were applied with default parameters unless specified otherwise. The Supplementary Tables were deposited in the Figshare (https://doi.org/10.6084/m9.figshare.28529096).

## Supporting information

Additional experimental details were included in the Supplementary Methods (DOCX). Alterations in the composition of gut multi-kingdom microorganisms (Figure S1), gut microbial functions at MetaCyc pathway levels (Figure S2), bacteriophages – bacterium interactions (Figure S3), a violin plot of 25 vital features associated with FBG (Figure S4), and alterations of targeted circulating metabolome profiles (Figure S5) (PDF). Summary of clinical parameters, dietary nutrient intakes and physical activities (Table S1-S3), information on sequencing data (Table S4), significantly altered gut multi-kingdom microorganisms and MetaCyc pathways (Table S5-S9), Spearman’s rank correlation analysis of gut intra-kingdom species and inter-kingdom species (Table S10-S15), viral host detection (Table S16), correlations among gut multi-kingdom microorganisms, serum metabolome, and phenome (Table S17-S20) (XLSX).

## Abbreviations

FBG: Fasting Blood Glucose
T2DM: Type 2 Diabetes
AAs: Amino Acids
FAs: Fatty Acids
SCFAs: Short-Chain Fatty Acids
MCFAs: Medium-Chain Fatty Acids
BAs: Bile Acids
BW: Body Weight
HR: Heart Ratio
WC: Waist Circumference
BMI: Body Mass Index
SBP: Systolic Blood Pressure
DBP: Diastolic Blood Pressure
HDL: High-Density Lipoprotein Cholesterol
LDL: Low-Density Lipoprotein Cholesterol
FFQ: Food Frequency Questionnaires
PAQ: Physical Activities Questionnaires
PCA: Principal Component Analysis
LME: Linear Mixed Model
rdcv-PLS: Partial Least Square-Discriminant Analysis Incorporated into a Repeated Double Cross-Validation Framework
PCoA: Principal Coordinates Analysis
PERMANOVA: Permutation Multivariate Analysis of Variance
OR: Odds Ratio
CI: Confidence Interval
KEGG: Kyoto Encyclopedia of Genes and Genomes
KOs: KEGG Orthologs
MetaPhlAn 4: Metagenomic Phylogenetic Analysis 4
HUMAnN 3: Human Microbiome Project Unified Metabolic Analysis Network 3
UHPLC-MS: Ultra High Performance Liquid Chromatography – Mass Spectrometer
GC-MS: Gas Chromatography – Mass Spectrometer
CRISPR: Clustered Regularly Interspaced Short Palindromic Repeat
MVP: Microbe Versus Phage
FMT: Fecal Microbiota Transplantation
FVT: Fecal Virome Transplantation
VLP: Viral-Like Particles
