# Supplementary material for: Moderate altitude exposure impacts extensive host-microbiota multi-kingdom connectivity with serum metabolome and fasting blood glucose
Source: Virulence. 2025 Jul 9;16(1):2530660. doi: 10.1080/21505594.2025.2530660 (PMC12269675; doi:10.1080/21505594.2025.2530660)
Supplement: Supplemental Material [file KVIR_A_2530660_SM2940.docx]

**Supplementary Methods**

**Site description**

Nyingchi city (29.5° N, 94.3° E) is known as "Little Switzerland" in China with no heavy industry nearby and tourism is the local economic pillar industry. It is located beside the Niyang River (one of the main tributaries of the Brahmaputra), and located in the southeastern Qinghai-Tibet Plateau and Tibet southeast gorge forest area, which accounts for 80% of the total forest area (1.47 × 10^7^ ha) in Tibet (1). The average annual temperature is 6 °C ~ 17 °C. The average annual sunshine duration is < 2100 hours. The average annual solar radiation is 5460 ~ 7530 MJ/m2. The average annual precipitation is 500 ~ 1000 mm. The average annual relative humidity is 60% ~ 75%. The average annual wind speed is 2 ~ 4 m/s. The climate is mainly dominated by the Indian Ocean and the Pacific warm current. Meteorological data of Nyingchi city were from Nyingchi Municipal People's Government Network, available online: <http://www.linzhi.gov.cn/linzhi/zmlz/qh.shtml>.

**Study cohort and sample collection**

The study protocol was approved by the Human Research and Ethics Committee of the People’s Hospital of Nyingchi and was registered at ChiCTR.org.cn (*ChiCTR1800016854*), which was performed in accordance with the principle of the Helsinki Declaration II. Each participant provided written informed consent.

Briefly, a total of 94 healthy individuals were recruited for this study comprised two groups. The first group (n=47) comprised healthy Han Chinese aid-Tibet volunteers (male/female=35/12; age, 40.37±6.06 years, mean ± s.d.) from Guangzhou city who entered Bayi District, Nyingchi city in July 2018 as short-term residents for 12 months. Anthropometric data and blood samples were collected at baseline and months 6 and 12 post-moderate-altitude exposure. Fecal samples were collected at baseline and months 12 post-moderate-altitude exposure. The second group (n=47) comprised healthy volunteers (Han Chinese population (group Local_H): n = 36; male/female=10/26; age, 30.86 ± 8.30 years (mean ± s.d.) and Tibetans population (group Local_T): n = 11; male/female=1/10; age, 35.73 ± 8.57 years (mean ± s.d.)) residing in Nyingchi for more than five years. Anthropometric data, blood samples and fecal samples were collected. The first group was applied to integrate analysis of gut multi-kingdom microorganisms, serum metabolites and clinical indices to demonstrate which clinical index was significantly associated with gut microbiota ecosystem and serum metabolites under moderate-altitude exposure. The second group was a pilot study of the effects of ethnicity and moderate-altitude exposure time on the gut microbiota ecosystem and serum metabolome.

In addition, a team of nutritionists along with aid-Tibet volunteers from Guangdong entered in Nyingchi and systematically planned the diet of volunteers to ensure the stability of nutrition components. The dietary intake and physical activity of these volunteers before and after entering Nyingchi were recorded as self-reported questionnaires (food frequency questionnaires (FFQ) and physical activities questionnaires (PAQ)). During the follow-up, parts of these healthy Han Chinese aid-Tibet volunteers left Nyingchi as the work arrangements and dropped out. Eligible subjects were healthy volunteers by being taken for disease inquiry and physical examination, and were identified with normal glucose tolerance according to the 1999 WHO criteria after oral glucose tolerance test. None of the study subjects had taken antibiotics, anti-obesity agents, hormones, or probiotics at least 1 month before the initiation of the study.

Blood samples for clinical chemistry analyses were collected after overnight fasting for at least 10 h. Serum samples were centrifuged and stored at −80 °C until analysis. Fecal samples were collected, frozen immediately, and transported to the laboratory within 2 hours and stored at -80 °C for analysis. In total, 83 fecal samples were subjected to metagenomics sequencing, 164 serum samples were subjected to AAs, FAs (SCFAs, MCFAs) and BAs measurements, respectively.

**DNA extraction and sequencing**

The fecal samples were snap-frozen in liquid nitrogen and stored at -80 °C. The fecal samples used for metagenomic sequencing were extracted using the sodium dodecyl sulphate (SDS) method. DNA was subsequently diluted to 1ng/μL using sterile ddH2O, and its degradation degree and contamination were assessed on 1% agarose gels. DNA purity (OD260/OD280) was determined using the NanoDrop Microvolume Spectrophotometer (Thermo Fisher Scientific, USA). DNA concentration was measured using the Qubit® dsDNA Assay Kit in Qubit® 2.0 Fluorometer (Life Technologies, Carlsbad, CA, USA). All samples were sequenced on Illumina NovaSeq 6000 platform generating approximately 10 Gbp of 150-bp paired end reads per sample.

**Amino acids measurements using ultra-high performance liquid chromatography - mass spectrometer (UHPLC-MS)**

As previously described (2), the serum samples were defrosted in an ice water bath and mixed in a vortex for 30 s. A 50 μL aliquot of each sample was precisely transferred to an Eppendorf tube. After the addition of 200 μL of extraction solution (acetonitrile-methanol, 1:1, with internal standard mixture (consisted of L-Tryptophan-(indole-d5), L-Methionine-13C,d3, 4-Aminobutyric acid-2,2,3,3,4,4-d6, L-Glutamate-13C5,15N, L-Serine-d3, L-Asparagic Acid-d3, L-Arginine-13C6, L-Lysine-d4 HCl), precooled at -20 °C), the samples were vortexed for 30 s and sonicated for 15 min in ice-water bath, followed by incubation at -40 °C for one hour and centrifugation at 12000 rpm and 4 °C for 15 min. Then the supernatants were separated with Agilent 1290 Infinity II series UHPLC System (Agilent Technologies), equipped with a Waters ACQUITY UPLC BEH Amide column (100 × 2.1 mm, 1.7 μm, Waters). The column was eluted with acetonitrile-water solution containing 1% formic acid. The column temperature was set at 35 °C. The auto-sampler temperature was set at 4 °C and the injection volume was 1 μL. MS analysis was carried out using Agilent 6460 triple quadrupole mass spectrometer (Agilent Technologies), equipped with an AJS electrospray ionization (AJS-ESI) interface. Typical ion source parameters were capillary voltage = +4000/-3500 V, Nozzle Voltage = +500/-500 V, gas (N2) temperature = 300 °C, gas (N2) flow = 5 L/min, sheath gas (N2) temperature = 250 °C, sheath gas flow = 11 L/min, nebulizer = 45 psi. For the analysis of human serum samples, the RSD for internal standards mixture in the QC samples was on average of 5.97%, 7.30%, 8.23%, 10.03%, 8.27%, 17.67%, 7.57% and 8.70%, respectively.

**Short-chain fatty acids and medium-chain fatty acids measurements using the gas chromatography - mass spectrometer (GC-MS)**

As previously described (3), 50 μL serum was mixed with 0.05 mL 50 % H_2_SO_4_ and 0.2 mL of 2-Methylvaleric acid (25 mg/L stock in methyl tert-butyl ether) as internal standard. The amalgamation was subjected to vortex mixing for 30 s, oscillations in 10 min, then ultrasound treated for 10 min with incubation in ice water. Subsequently, centrifugation was conducted for 15 min at 10000rpm, 4 °C. Keep at −20 °C for 30 min, the organic phase was collected and analyzed using SHIMADZU GC2030-QP2020 NX gas chromatography-mass spectrometer (Shimadzu Corporation, Kyoto, Japan) equipped with a HP-FFAP capillary column (30m×250μm×0.25μm, Agilent Technologies, Wilmington, DE, USA). A 1 μL aliquot of the analyte was injected in split mode (5:1). Helium was used as the carrier gas, with a front inlet purge flow of 3 mL min^−1^, and a gas flow rate through the column was 1 mL min^−1^. The initial temperature was maintained at 80 °C for 1 min, subsequently elevated to 200 °C at a rate of 10 °C min^−1^ for 5 min, and then sustained for 1 min at 240 °C at a rate of 40 °C min^−1^. The injection, transfer line, quad and ion source temperatures were 240 °C, 240 °C, 200 °C and 150 °C, respectively. The energy was -70 eV in electron impact mode. The mass spectrometry data were acquired in Scan/SIM mode with the m/z range of 33-150 after a solvent delay of 3.5 min. The RSD for internal standard in the QC samples averaged at 6.24% during the analysis of human serum samples.

**Bile acids measurements using the UHPLC-MS/MS**

As previously described (4), 50 μL of plasma sample was extracted with 200 μL acetonitrile: methanol (1:1, v:v) containing isotopically-labelled internal standard mixture (Taurochenodeoxycholic acid-d4 sodium salt, Glycocholic acid-d4, Deoxycholic acid-d6), the samples were vortexed for 30 s, sonicated for 10 min in ice-water bath followed by incubation at -40 ^o^C for 1 h. After centrifugation at 12000 rpm for 15 min at 4 ^o^C, the supernatant was transferred into a new microcentrifuge tube for UHPLC-MS/MS analysis. Bile acids were measured using a Vanquish UHPLC System (Thermo Fisher Scientific, SanJose, CA), and separated with BEH C18 column (150 × 2.1 mm, 1.7 μm, Waters, Manchester, UK) using acetonitrile-water solution containing 1 mmol/L ammonium acetate and 1 mmol/L acetic acid in parallel reaction monitoring (PRM) mode. Q Exactive HFX mass spectrometer (Thermo Fisher Scientific, SanJose, CA) was applied for assay development in negative mode. The following ion source parameters were applied: spray voltage = -3100 V, sheath gas (N_2_) flow rate = 40, aux gas (N_2_) flow rate = 15, sweep gas (N_2_) flow rate = 0, aux gas (N_2_) temperature = 350 ^o^C, capillary temperature = 320 ^o^C. Moreover, PRM data was acquisited and processed by Xcalibur Software (*Version 4.1*, Thermo Fisher Scientific). Finally, the concentration of bile acids was calculated according to the calibration curve of standard substance, and bile acids present in more than 10% of the participants were included in subsequent analyses as previously described (5). For the analysis of human serum samples, the RSD for internal standards mixture in the QC samples was on average of 3.78%, 2.26% and 3.17%, respectively.

**Metagenomic sequencing analysis**

***Bacteria taxonomic profiling****.* Adapter was trimmed and low-quality reads were filtered using Trimmomatic (v0.39). Then, host sequences were removed by aligning sequencing reads back to the host genome reference (hg38) using SOAP2 (v2.20) when sequence identity exceeds 90% (6). Taxonomic profiling of the metagenomic samples was performed using metagenomic phylogenetic analysis 4 (MetaPhlAn 4, v4.0.6) (7), which uses clade-specific markers to provide pan-microbial (bacterial, archaeal and eukaryotic) quantification at species-level. MetaPhlAn was run with parameters '--read_min_len 50 --ignore_eukaryotes --ignore_archaea'.

***Fungi and archaea taxonomic profiling****.* The quality filtered reads were taxonomically classified using Kraken 2 (v2.1.3) (8) using the RefSeq database (Fungi or archaea) as a reference with default parameters. The species abundance estimation was improved using Bracken (v2.6.2) (9). The counts were normalized to 100%.

***B*acteriophages *taxonomic profiling****.* The reads in each sample were assembled with SPAdes assembler (v3.15) (10) in metagenomic mode. The contigs with length < 1 kb were discarded. The contigs constructed from the viral fraction were screened with a gene enrichment-based method VirSorter 2 (v2.2) (11) and a k-mer frequency-based method VirFinder (v1.1) (12) to identify and remove bacteria-like contigs. VirSorter2 was performed using max_score > 0.95 as a threshold. VirFinder was performed using a default prediction model, and *P* < 0.05 with score > 0.7 as a threshold. CD-HIT (v.4.8) (13) was used to cluster pooled contigs (non-redundant contigs) at 95% global average nucleotide identity (-c 0.95). We classified viral contigs using viral RefSeq genomes and protein databases (from NCBI). To improve the viral protein homology search classification of viral contigs, we additionally used the phage structural proteins in the PfamA annotation with an *E* value < 1e-10. Finally, family level taxonomic annotations were assigned to the contigs that were not classified in the previous step using Demovir script with default parameters and database (14). We used the clustered regularly interspaced short palindromic repeat (CRISPR) Recognition Tool (v1.6.0) (15) to predict the CRISPR spacers sequences of bacterial genomes (Refseq reference genomes) and aligned the CRISPR spacers sequences with virus contigs. Only when the host CRISPR spacers completely matched the virus contigs (100% sequence identity of the entire CRISPR spacers), we assigned the host to the contigs. The unaligned contigs were further aligned with the Microbe Versus Phage (MVP) database (16) to predict the possible hosts of the virus contigs. Finally, we combined the prediction results of the two methods.

***Microbial functional profiles****.* Gene abundance profile was calculated as previously described (17). Functional profiling was performed by Human Microbiome Project Unified Metabolic Analysis Network 3 (HUMAnN 3, v3.6.1) pipeline (18) using the search mode of UniProt Reference Clusters (uniref90) and pathway MetaCyc, which contains the functional potential of each metagenome sample. Sample reads are mapped against this database to quantify gene presence and abundance on a per-species basis. A translated search is then performed against a UniRef-based protein sequence catalogue for all reads that fail to map at the nucleotide level. The results are abundance profiles of gene families (UniRef90s), stratified by each species contributing those genes, and which can then be summarized to higher-level gene groupings such as ECs or KOs.

**References**

1. Wang W, Xu W, Wen Z, Wang D, Wang S, Zhang Z, Zhao Y, Liu X. 2019. Characteristics of Atmospheric Reactive Nitrogen Deposition in Nyingchi City. Sci Rep 9:4645.

2. Wang Z, Li B, Li S, Lin W, Wang Z, Wang S, Chen W, Shi W, Chen T, Zhou H, Yinwang E, Zhang W, Mou H, Chai X, Zhang J, Lu Z, Ye Z. 2022. Metabolic control of CD47 expression through LAT2-mediated amino acid uptake promotes tumor immune evasion. Nat Commun 13:6308.

3. Hou Y, Zhang Z, Cui Y, Peng C, Fan Y, Tan C, Wang Q, Liu Z, Gong J. 2022. Pu-erh tea and theabrownin ameliorate metabolic syndrome in mice via potential microbiota-gut-liver-brain interactions. Food Res Int 162:112176.

4. Han J, Liu Y, Wang R, Yang J, Ling V, Borchers CH. 2015. Metabolic profiling of bile acids in human and mouse blood by LC-MS/MS in combination with phospholipid-depletion solid-phase extraction. Anal Chem 87:1127-1136.

5. Chen L, van den Munckhof ICL, Schraa K, Ter Horst R, Koehorst M, van Faassen M, van der Ley C, Doestzada M, Zhernakova DV, Kurilshikov A, Bloks VW, Groen AK, Human Functional Genomics P, Riksen NP, Rutten JHW, Joosten LAB, Wijmenga C, Zhernakova A, Netea MG, Fu J, Kuipers F. 2020. Genetic and Microbial Associations to Plasma and Fecal Bile Acids in Obesity Relate to Plasma Lipids and Liver Fat Content. Cell Rep 33:108212.

6. Qin J, Li Y, Cai Z, Li S, Zhu J, Zhang F, Liang S, Zhang W, Guan Y, Shen D, Peng Y, Zhang D, Jie Z, Wu W, Qin Y, Xue W, Li J, Han L, Lu D, Wu P, Dai Y, Sun X, Li Z, Tang A, Zhong S, Li X, Chen W, Xu R, Wang M, Feng Q, Gong M, Yu J, Zhang Y, Zhang M, Hansen T, Sanchez G, Raes J, Falony G, Okuda S, Almeida M, LeChatelier E, Renault P, Pons N, Batto JM, Zhang Z, Chen H, Yang R, Zheng W, Li S, Yang H, Wang J, Ehrlich SD, Nielsen R, Pedersen O, Kristiansen K, Wang J. 2012. A metagenome-wide association study of gut microbiota in type 2 diabetes. Nature 490:55-60.

7. Blanco-Miguez A, Beghini F, Cumbo F, McIver LJ, Thompson KN, Zolfo M, Manghi P, Dubois L, Huang KD, Thomas AM, Nickols WA, Piccinno G, Piperni E, Puncochar M, Valles-Colomer M, Tett A, Giordano F, Davies R, Wolf J, Berry SE, Spector TD, Franzosa EA, Pasolli E, Asnicar F, Huttenhower C, Segata N. 2023. Extending and improving metagenomic taxonomic profiling with uncharacterized species using MetaPhlAn 4. Nat Biotechnol 41:1633-1644.

8. Wood DE, Lu J, Langmead B. 2019. Improved metagenomic analysis with Kraken 2. Genome Biol 20:257.

9. Lu J, Breitwieser FP, Thielen PM, Salzberg SLJb. 2016. Bracken: Estimating species abundance in metagenomics data.

10. Nurk S, Meleshko D, Korobeynikov A, Pevzner PA. 2017. metaSPAdes: a new versatile metagenomic assembler. Genome Res 27:824-834.

11. Guo J, Bolduc B, Zayed AA, Varsani A, Dominguez-Huerta G, Delmont TO, Pratama AA, Gazitua MC, Vik D, Sullivan MB, Roux S. 2021. VirSorter2: a multi-classifier, expert-guided approach to detect diverse DNA and RNA viruses. Microbiome 9:37.

12. Ren J, Ahlgren NA, Lu YY, Fuhrman JA, Sun F. 2017. VirFinder: a novel k-mer based tool for identifying viral sequences from assembled metagenomic data. Microbiome 5:69.

13. Li W, Godzik A. 2006. Cd-hit: a fast program for clustering and comparing large sets of protein or nucleotide sequences. Bioinformatics 22:1658-1659.

14. Shkoporov AN, Clooney AG, Sutton TDS, Ryan FJ, Daly KM, Nolan JA, McDonnell SA, Khokhlova EV, Draper LA, Forde A, Guerin E, Velayudhan V, Ross RP, Hill C. 2019. The Human Gut Virome Is Highly Diverse, Stable, and Individual Specific. Cell Host Microbe 26:527-541 e525.

15. Russel J, Pinilla-Redondo R, Mayo-Munoz D, Shah SA, Sorensen SJ. 2020. CRISPRCasTyper: Automated Identification, Annotation, and Classification of CRISPR-Cas Loci. CRISPR J 3:462-469.

16. Gao NL, Zhang C, Zhang Z, Hu S, Lercher MJ, Zhao XM, Bork P, Liu Z, Chen WH. 2018. MVP: a microbe-phage interaction database. Nucleic Acids Res 46:D700-D707.

17. Li J, Jia H, Cai X, Zhong H, Feng Q, Sunagawa S, Arumugam M, Kultima JR, Prifti E, Nielsen T, Juncker AS, Manichanh C, Chen B, Zhang W, Levenez F, Wang J, Xu X, Xiao L, Liang S, Zhang D, Zhang Z, Chen W, Zhao H, Al-Aama JY, Edris S, Yang H, Wang J, Hansen T, Nielsen HB, Brunak S, Kristiansen K, Guarner F, Pedersen O, Dore J, Ehrlich SD, Meta HITC, Bork P, Wang J, Meta HITC. 2014. An integrated catalog of reference genes in the human gut microbiome. Nat Biotechnol 32:834-841.

18. Beghini F, McIver LJ, Blanco-Miguez A, Dubois L, Asnicar F, Maharjan S, Mailyan A, Manghi P, Scholz M, Thomas AM, Valles-Colomer M, Weingart G, Zhang Y, Zolfo M, Huttenhower C, Franzosa EA, Segata N. 2021. Integrating taxonomic, functional, and strain-level profiling of diverse microbial communities with bioBakery 3. Elife 10.
