# Supplementary figures and images for: Moderate altitude exposure impacts extensive host-microbiota multi-kingdom connectivity with serum metabolome and fasting blood glucose
Source: Virulence. 2025 Jul 9;16(1):2530660. doi: 10.1080/21505594.2025.2530660 (PMC12269675; doi:10.1080/21505594.2025.2530660)

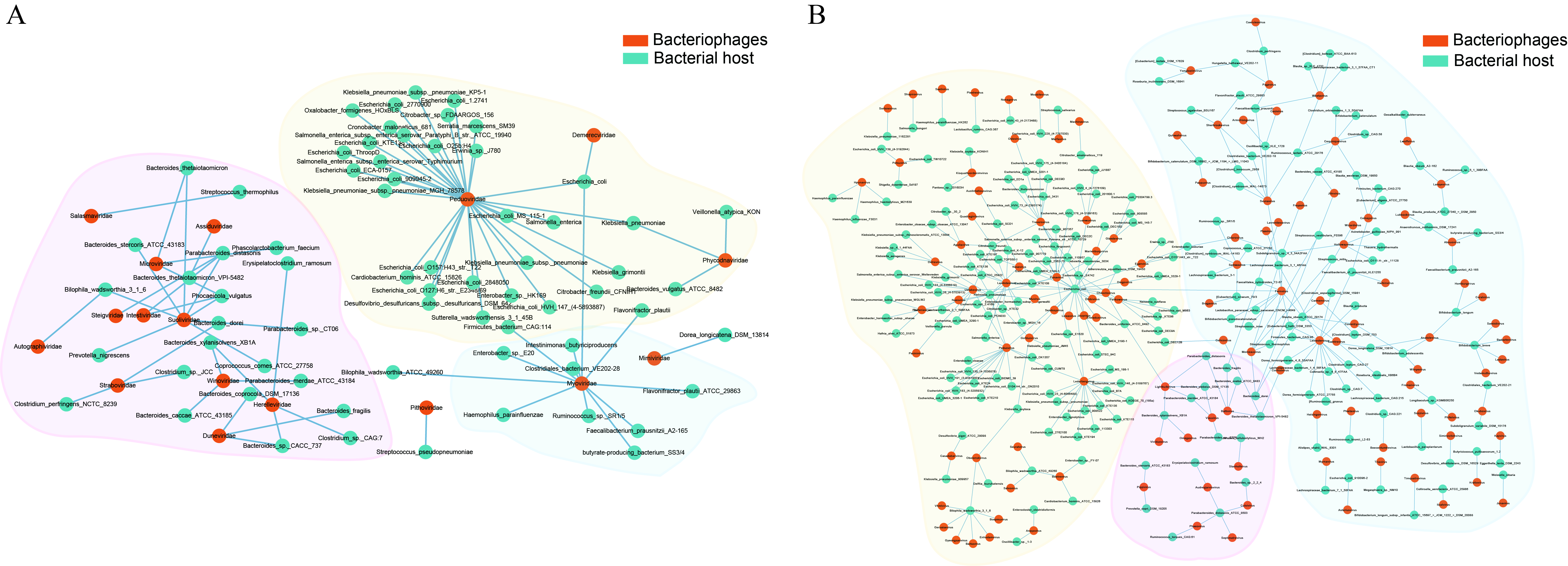

Supplement: Figure S3.tif [file KVIR_A_2530660_SM2258.tif]

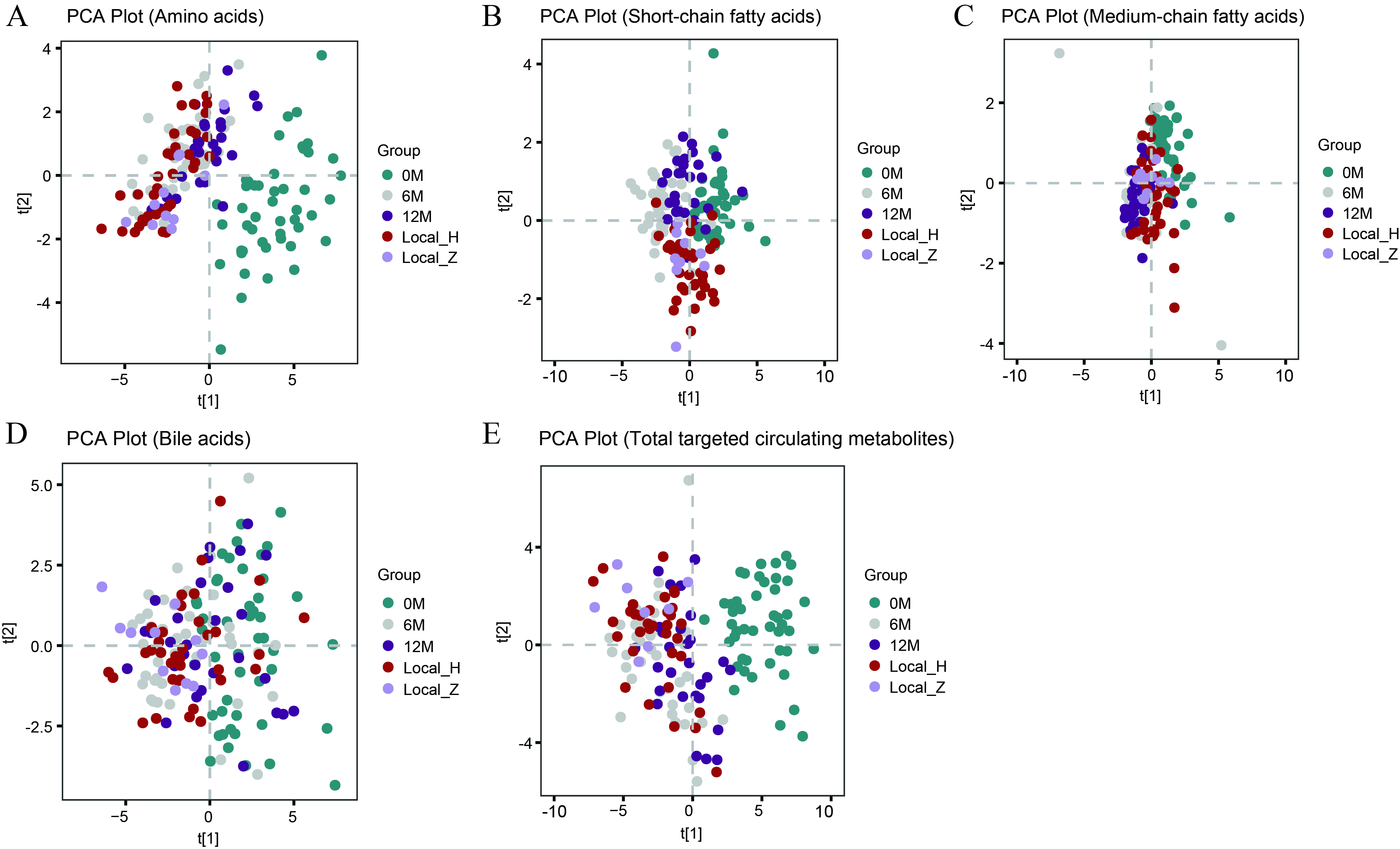

Supplement: Figure S5.tif [file KVIR_A_2530660_SM2257.tif]

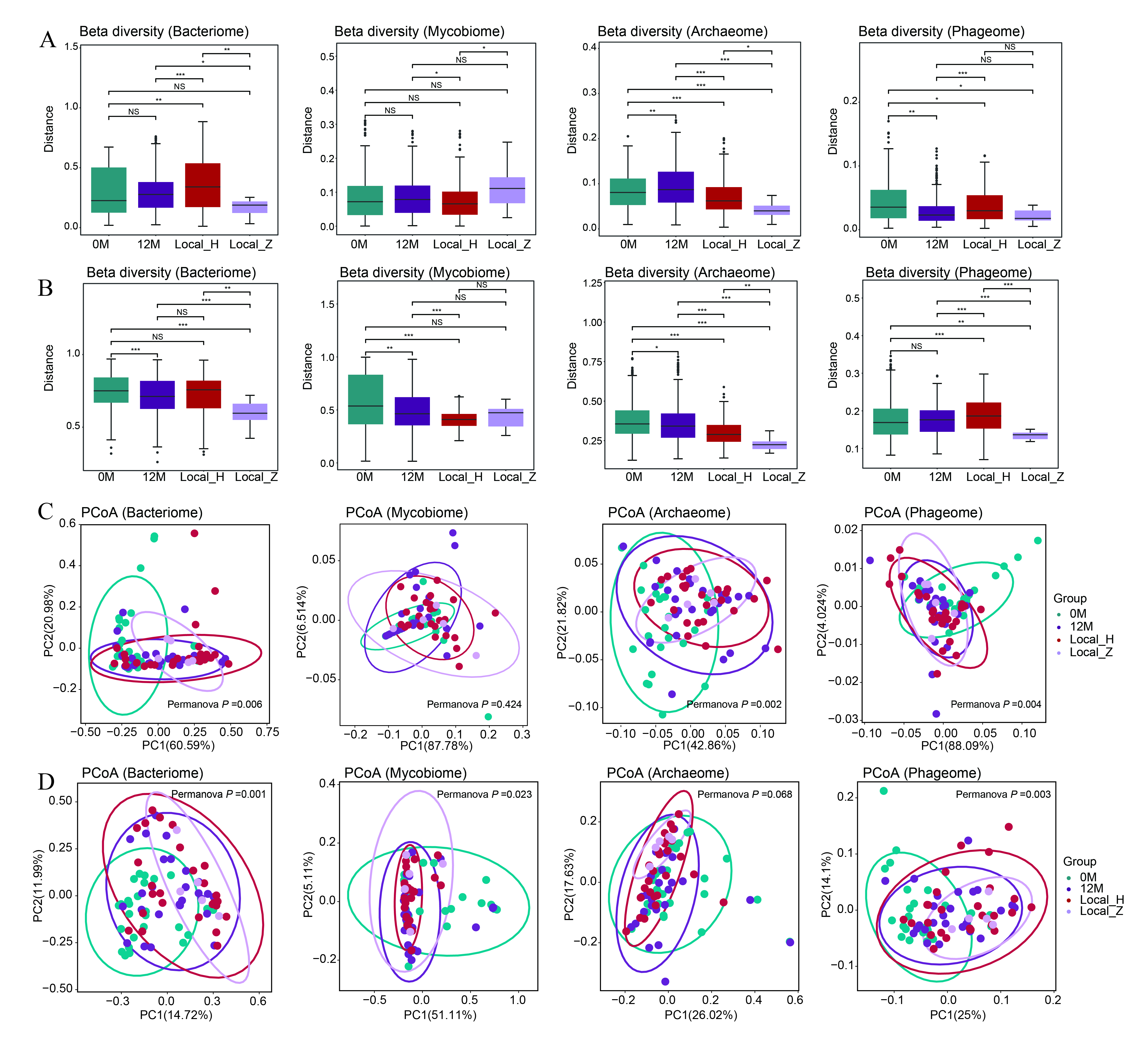

Supplement: Figure S1.tif [file KVIR_A_2530660_SM2256.tif]

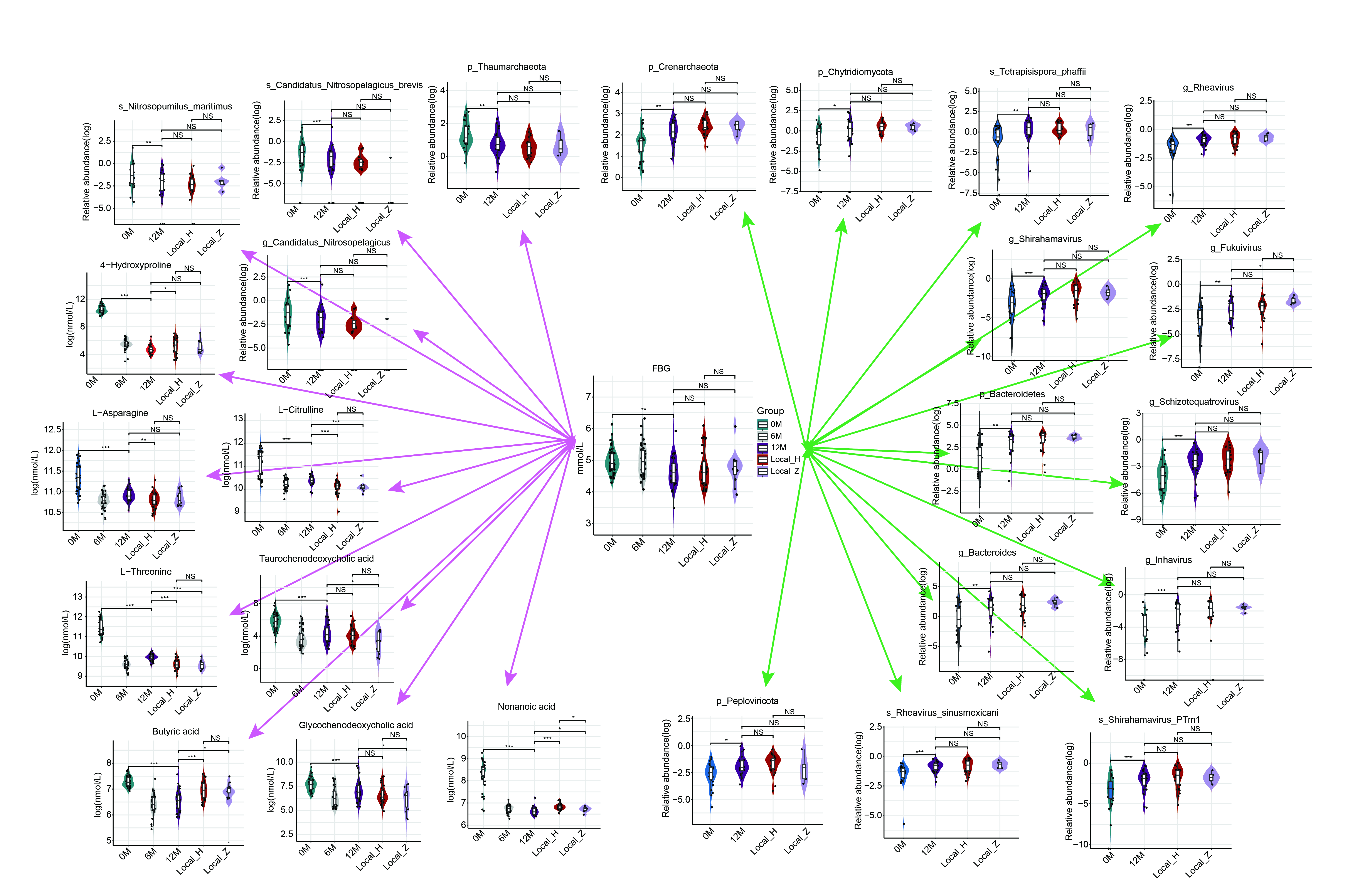

Supplement: Figure S4.tif [file KVIR_A_2530660_SM2255.tif]
